# Reduction of Contaminants (Physical, Chemical, and Microbial) in Domestic Wastewater through Hybrid Constructed Wetland

**DOI:** 10.1155/2013/350260

**Published:** 2013-05-02

**Authors:** Shama Sehar, Rabia Aamir, Iffat Naz, Naeem Ali, Safia Ahmed

**Affiliations:** Microbiology Research Laboratory, Department of Microbiology, Quaid-i-Azam University, Islamabad 45320, Pakistan

## Abstract

The current research was focused mainly on the designing and construction of efficient laboratory scale hybrid constructed wetland (HCW) for the treatment of domestic wastewater. Parameters like COD, BOD_5_, PO_4_, SO_4_, NO_3_, NO_2_, and pathogenic indicator microbes were monitored after hydraulic retention time (HRT) of 4, 8, 12, 16, and 20 days. Treatment efficiency of HCW kept on increasing with the increase in hydraulic retention time. Maximum efficiency of HCW was observed with a 20-day HRT, that is, 97.55, 97.5, 89.35, 80.75, 96.04, 91.52, and 98.6% reduction from the zero time value for COD, BOD_5_, PO_4_, SO_4_, NO_3_, NO_2_, and fecal coliforms, respectively. After 20 days' time, the treated water was free of almost all nutrients and microbial pollutants. Hence, increasing hydraulic retention time was found to ameliorate the operational competence of HCW. Thus HCW can serve as a promising technology for wastewater treatment and can be scaled up for small communities in the developing countries.

## 1. Introduction

Water is extremely essential for the survival of all living organisms. Like many other developing countries, Pakistan is also regarded as a water-stressed country, and it is likely to have a water scarcity in the near future [1, 2]. The quality and quantity of fresh water is deteriorated by the discharge of untreated municipal wastewater, and according to a recent report, only 12% of the urban wastewater is treated in municipal treatment plants [3]. The exploitation of raw wastewater is risky both from environmental and health perspectives, mainly because it contains biodegradable organic and inorganic matter, toxic substances, and disease-causing agents [4]. 

In the recent years, diverse technologies have been introduced for the treatment of municipal, domestic, industrial, and nuclear wastewater. The fundamental procedures being used in these innovative wastewater treatment technologies are of physical, chemical, and biological in nature [5]. In biological technologies, constructed wetlands (CWs) for wastewater treatment have the merits of minimum operational cost, ease in management, and environment friendly features [6], and they are the most recommended system for small communities [7]. CWs are artificially designed system composed of a medium such as soil or gravel planted with vegetation tolerant of saturated soil conditions [8] which is equally effective for pathogens, organic, and toxic metals [9]. The removal efficiency of CWs can be tuned by altering hydraulic retention time and temperature [10]. 

Most hybrid systems consist of vertical flow (VF) and horizontal flow (HF) systems arranged in a staged manner [11]. In hybrid constructed wetlands, VF and HF complement each other and thus make the system more proficient. Therefore it is possible to produce wastage whose biological oxygen demand is low and that is partially denitrified and completely nitrified. Thus, the overall nitrogen would be much reduced in the final effluent [12]. 

The present research is focused on the designing and construction of lab scale hybrid constructed wetland (HCW) for effective treatment of domestic wastewater from residential colony, Quaid-i-Azam University, Islamabad, as this wastewater ultimately discharged into Rawal lake and pollutes it.

## 2. Methods

### 2.1. Designing of Hybrid Constructed Wetland (HCW)

**Figure 1:**
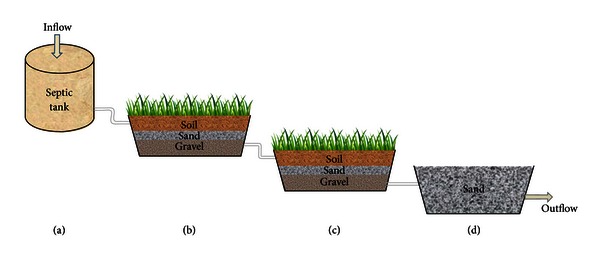
Schematic representation of hybrid constructed wetland (HCW) with (a) septic tank for primary sedimentation of untreated wastewater, (b) 1st vegetative unit with subsurface vertical flow (SS-VF), (c) 2nd vegetative unit with subsurface horizontal flow (SS-HF), and (d) sand bed (Sb) for final polishing of treated wastewater.

A small-scale HCW was comprised of one septic tank (500 L) and three rectangular operational units (length × width × height = 4 × 1.5 × 1.5 feet). The first two treatment units (SS-VF and SS-HF) were composed of three layers one over the other (organic soil 12.5 cm, sand 15 cm, and gravel 7.5 cm). These two units were planted with *Paspalidium flavidum*, the common grass found growing naturally in sewage contaminated natural stream and tolerant enough for contaminants. The third unit consisted of a bed of sand (Sb) that served the purpose of removing suspended particles from the water that received treatment from the first two units. The four units were sequentially placed and interconnected by polyvinylchloride pipes (length = 125 inches, inner diameter = 2 cm) and were positioned with decreasing heights (1 feet) in order to facilitate the natural flow of water under gravitational pull. Valves and nozzles were used to regulate the flow rate of water from one unit to another. Two units (SS-VF and SS-HF), containing soil, sand, and gravel, were kept soaked with fresh water for 3 to 4 weeks in order to acquire saturated growth of grass and associated microbial community in rhizosphere, sand, and gravel bed before the start of experiment. This helped in the establishment of a compact bed suitable for wastewater treatment. It was run under different treatment times (4, 8, 12, 16, and 20 days) (continuously operated from April, 2011 to July, 2012 in Islamabad, PK). Temperature was continuously monitored during the study by using thermometer and was found to be in the range of 30–45°C. A schematic representation of the overall treatment process is shown in Figure 1.

### 2.2. Bacterial Profiling of Rhizosphere of *Paspalidium flavidum* Planted in HCW

Bacterial diversity colonizing the rhizosphere of *Paspalidium flavidum *planted in HCW was studied. For this purpose, 1 g soil sample was collected and different dilutions (10^−3^, 10^−5^, 10^−7^) were made. From these dilutions, 0.1 mL inoculum was inoculated onto nutrient agar plates using the spread plate method, and plates were incubated for 24 hours at 37°C. After incubation, different types of colonies on nutrient agar plates were distinguished on the basis of morphology. To obtain pure cultures different colonies were further subcultured on Eosin-methylene blue agar (EMB), *Salmonella-Shigella *agar (SSA), mannitol salt agar (MSA), *Pseudomonas* cetrimide agar (PCA), Blood agar (BA), and MacConkey's agar (MacA), and these plates were again incubated at 37°C for 24–36 hrs. Identification of subcultured organisms was carried out on the basis of plate morphological characteristics, microscopy, and biochemical tests.

### 2.3. Treatment of Domestic Wastewater

Wastewater was characterized soon after its collection and given a retention time of almost 3 hours in the septic tank for sedimentation of any particulate material and suspended solids. This partially treated wastewater was then passed through the subsequent three treatment units (SS-VF, SS-HF, and Sb) of HCW by giving a retention time of 4, 8, 12, 16, and 20 days. Temperature was constantly monitored throughout the operational phase of the hybrid CW. Water samples were collected from each processing unit and were analyzed through different physicochemical and microbiological tests. 

### 2.4. Physicochemical Analysis

Physico-chemical analysis of wastewater was carried out by determining different parameters; that is, pH (D-25 Horiba), electrical conductivity (EC) (WTWcind330i) and DO (MM-60R, TOA-DKK) were determined by their respective digital meters. Biochemical oxygen demand (BOD) was estimated by 5-day BOD test (5210 B standard method) and chemical oxygen demand (COD) by kit method; high range 14541 and low range 14560 CSB/COD kits (Merck, Germany). Standard method 1540 C, 2540 D was used to estimate total dissolved solids (TDS), total suspended solids (TSS) in water samples, respectively, Standard method 4500-P, 0375 Barium chromate, 4500 NO_3_-N, and 4500 NO_2_-N were used to determined orthophosphate, sulfates, nitrates, and nitrites, respectively, in water sample [13]. The reagents used for the analysis were AR grade, and instruments were of limit of precise accuracy.

### 2.5. Microbiological Analysis

Microbiological analysis of sewage was carried out by colony forming unit (CFU/mL) and most probable number technique (MPN index) before and after treatment through hybrid constructed wetland. These wastewater samples were serially diluted in sterile water up to 10^−10^. The dilutions were spread plated on NA plates, incubated for 24 hrs at 37°C. The colonies appeared were enumerated by colony counter, and estimations were made according to formulae CFU/mL = number of colonies × dilution factor/inoculum size. 

For the examination and enumeration of fecal coliforms, pathogens and other coliforms (*E. coli*, *Salmonella*, *Shigella*, *Klebsiella *sp., *Enterobacter*, and *Citrobacter*), untreated, and treated wastewater samples were incubated at 42.2°C for 24–48 hrs in MacConkey's broth using multiple tube technique having inverted Durham tubes. Positive tubes were subcultured on MacA, NA, and MSA plates and incubated at 37 ± 2°C for 24–48 hrs. Positive isolates (showing growth) were confirmed by microscopy, that is, gram's staining, and checked for total count. 

### 2.6. Statistical Analysis

Statistical analysis of each treatment was carried out using Microsoft Excel Program. In order to find percentage treatment, the treated sewage samples were compared by *t*-test, and *P* < 0.05 was considered as minimum value for statistical significance.

## 3. Results and Discussion

### 3.1. Bacterial Profiling of Rhizosphere of *Paspalidium flavidum*

*Paspalidium flavidum* was found growing naturally in sewage contaminated areas and was highly tolerant to harmful contaminants present in domestic wastewater. Selection of this plant was made on the basis of dense roots and root hair structure that serves as an important reservoir for accumulation of various microorganisms and thus helps in wastewater clarification, allowing good microbial growth. On the basis of microscopic, morphological, and biochemical tests, bacterial strains were identified according to Bergey's Manual of Determinative Bacteriology [14] as *Proteus *sp.*, Klebsiella *sp.*, Escherichia coli, Shigella *sp.*, Alcaligenes faecalis, Salmonella *sp.*, Enterobacter *sp.*, Bacillus *sp.*, Pseudomonas *sp.*, Micrococcus *sp., and* Staphylococcus *sp.from the rhizosphere soil sample (Table 1).

### 3.2. Analysis of Wastewater before Treatment

The quality of domestic wastewater was examined in triplicates. Apparently, it was grey in color with mordant smell. Parameters such as pH (7.91), NO_2_ (0.059 mg/L), NO_3_ (2.83 mg/L), PO_4_ (0.197 mg/L), SO_4_ (0.095 mg/L), TDS (480 mg/L), EC (510 *μ*S/cm), and Cl (35.87 mg/L) of the domestic wastewater were within the standard limits of WHO and US-EPA. However, TSS (478 mg/L), DO (2.5 mg/L), BOD_5_ (134.83 mg/L), and COD (199.23 mg/L) were considerably deviated from their prescribed limits, indicating the high level of contamination.

### 3.3. Analysis of Wastewater after Treatment at Different HRT

#### 3.3.1. Odour and pH

**Figure 2:**
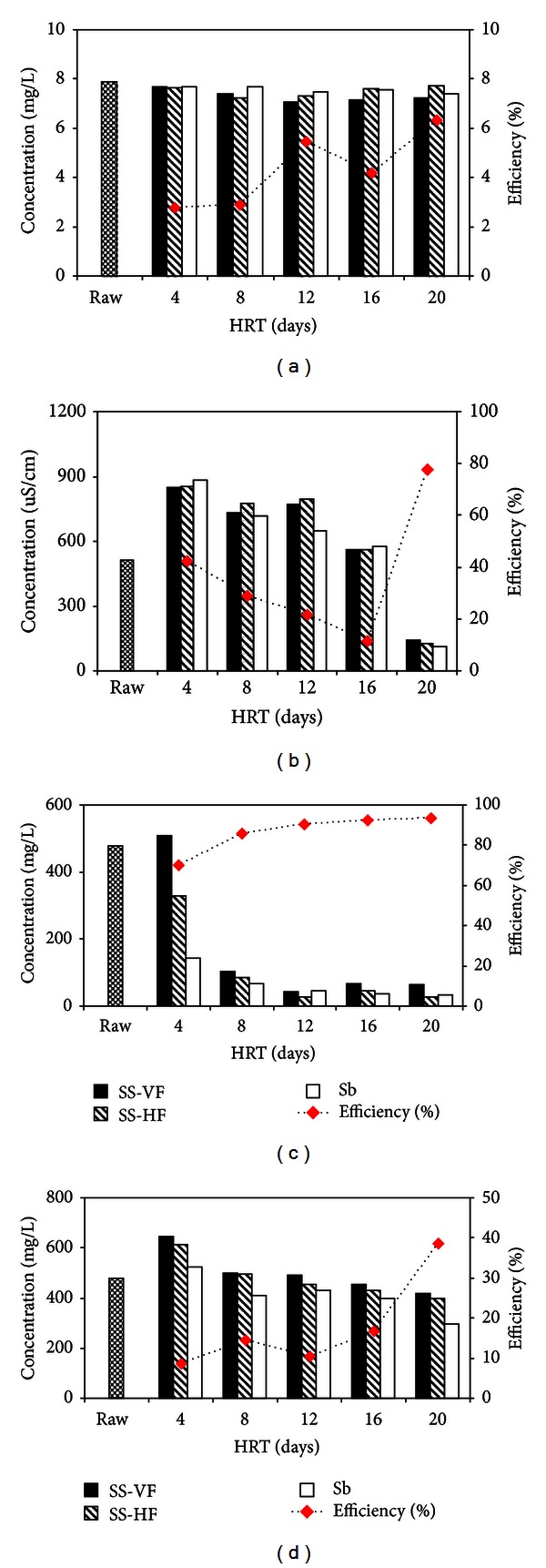
Change in concentration and % efficiency during treatment after giving different HRT. (a) pH, (b) electrical conductivity (EC), (c) total suspended solids (TSS), and (d) total dissolved solids (TDS).

Among physical parameters, odour is very important for the determination of water quality. In the present study, the untreated wastewater had unpleasant and penetrating smell. According to WHO standards, the clean water should be free of any type of odour. The main causative agent of foul odour is excessive growth of microbes, algal, and plant growth in profoundly polluted waters with high level of nutrients. In the degradation process of these plants and algae, bacteria produce a wide variety of unpleasant odours such as methane, rancid, and sulphur, and so forth [15]. In the hybrid CW, the odour of water was completely removed with an efficiency of 100% in all the treatments. The notable mineralization of the organic substances and removal of microbes during various treatments in the hybrid system resulted in the elimination of odour. In addition, pH value of the CW should be above 6 in order to achieve an efficient denitrification activity [16]. There was no profound effect on the pH values throughout the experimental period and the pH of the untreated and treated domestic wastewater remained within a range of 7.05–7.91 (Figure 2(a)). 

#### 3.3.2. Electrical Conductivity and Removal of Solids

The value of EC, TSS, and TDS were found to be 510 *μ*S/cm, 478, and 480 mg/L, respectively, in the untreated samples of colony wastewater. According to [17], the prescribed value of EC, TSS, and TDS in drinking water is 400–1215 *μ*S/cm, 25–80 mg/L and <1000 mg/L, respectively. In this study, it was found that EC value of untreated wastewater decreased gradually during treatment due to the decrease in TDS and TSS levels as EC is directly dependent on the suspended and dissolved solids [18]. This decrease in EC might be related to the conversion of NO_3_ into diatomic molecular nitrogen (N_2_), which also decreases EC levels of domestic wastewater, that is, 113 mg/L (77.84%) with HRT of 20 days (Figure 2(b)). TSS concentration reduced to 144, 67, 47, and 37 with HRT of 4, 8, 12, and 16 days, respectively. The maximum reduction of 32 mg/L (93.30%) was observed after 20 days that showed highly significant treatment (*P* < 0.001) (Figure 2(c)). On the other hand, TDS showed initial increase in its value, but with the increase in HRT, it decreased to maximum reduction after 20-day HRT (295 mg/L) (Figure 2(d)).

#### 3.3.3. Removal of Organic Pollutants

**Figure 3:**
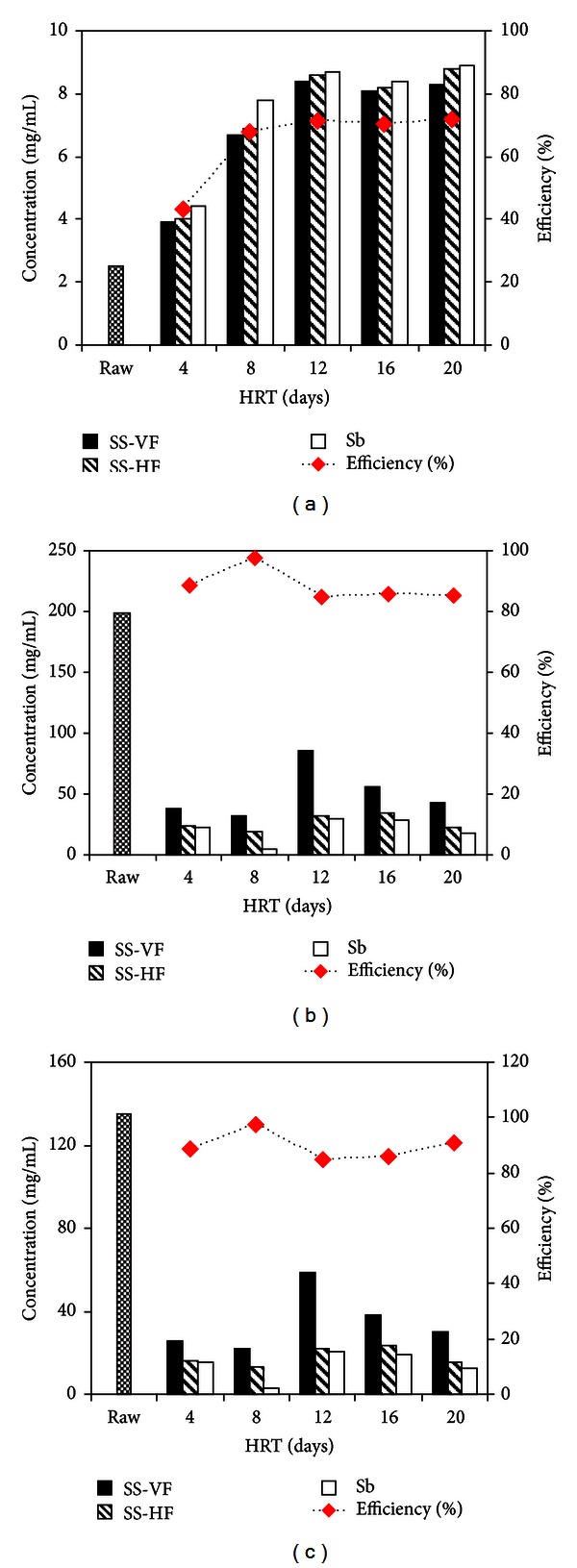
Change in concentration and % efficiency during treatment after giving different HRT. (a) Dissolved oxygen (DO), (b) chemical oxygen demand (COD), and (c) biological oxygen demand (BOD_5_).

Organic pollutants include biological and chemical oxygen demand and are interrelated with the amount of dissolved oxygen. Dissolved oxygen is an important parameter in water quality assessment and reflects the physical and biological processes prevailing in the water bodies [19]. The concentration of DO in raw water sample was very low, that is, 2.5 mg/L, and according to WHO, the prescribed limit of DO for drinking water is 6–8 mg/L [17]. A noteworthy improvement in the quality of water was observed in terms of DO with subsequent increase in hydraulic retention time until it reached the maximum up to 8.9 mg/L (71.90%) in the final treatment of HRT of 20 days (Figure 3(a)). DO value as high as 8.7 mg/L was also reported [20]. The DO values as high as 7.1 ± 0.8 mg/L of wastewater mean that this water could support the oxygen requirements of the aquatic organisms [21]. One of the reasons of the noteworthy increased DO in the vegetative units of the hybrid CW might be the biodegradation of compounds present in wastewater that previously used dissolved oxygen for various oxidation-reduction reactions and thus the release of oxygen through roots into the rhizophore [22]. 

The untreated (raw) wastewater had high range of COD and BOD_5_ (199.23 and 134.83 mg/L) as compared to US-EPA standards, that is, (5–8 mg/L and 8–10 mg/L), respectively [23]. These high values were due to the presence of large amount of organic compounds in the domestic wastewater. Maximum activity was observed with 8-day HRT where both COD and BOD values were reduced up to 97.5% (4.8 and 3.25 mg/L resp.) and showed highly significant (*P* < 0.001) treatment (Figures 3(b) and 3(c)). Akratos and Tsihrintzis [24] also found COD and BOD_5_ removals of 91 and 90.1%, respectively, with HRT of 8-days and there was no significant improvement in their reduction by increasing the HRT (i.e., 16 and 20 days). This decrease in BOD5 and COD values might be due to high biodegradation of organic contaminants of wastewater during constant microbial activities in the rhizosphere of *Paspalidium flavidum *having dense root hair structure.

#### 3.3.4. Nutrient Removal

**Figure 4:**
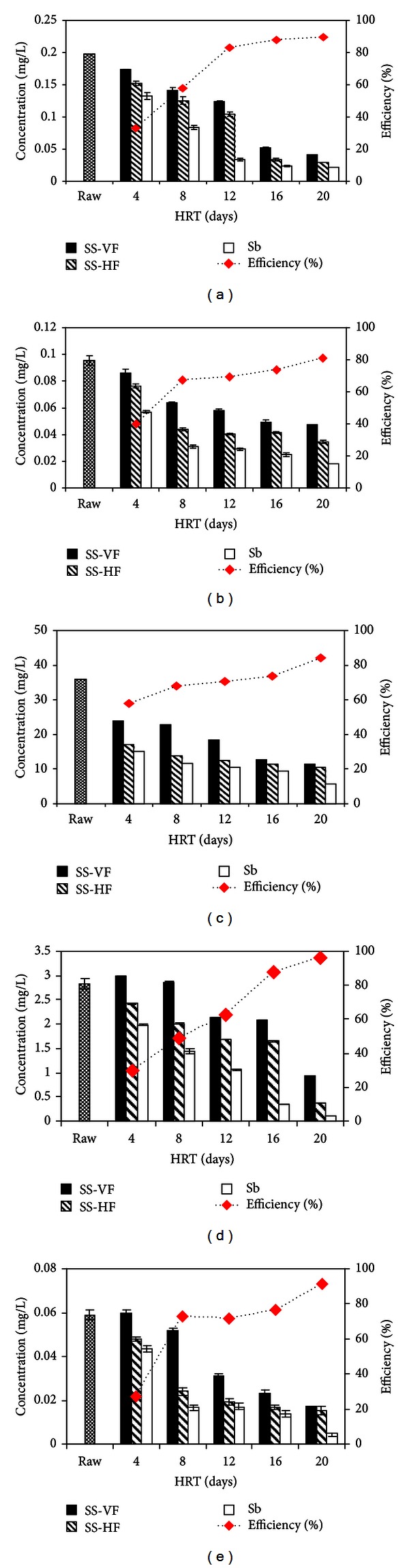
Concentration of different nutrients and % efficiency during treatment by giving different HRT in hybrid constructed wetland; (a) phosphates (b) sulphates (c) chlorides (d) nitrates and (e) nitrites.

An effect of hydraulic retention time on various nutrients (phosphate, sulphate, chlorides, nitrate, and nitrite) was observed. Phosphates are considered as one of the major nutrients, and they enter in the form of polyphosphates into sewage from detergents, animal, and human excreta, and so forth, and are source of eutrophication in receiving water bodies. They are removed from wastewater by the intracellular microbial accumulation for cellular activities and biomass production [25]. No prescribed values are defined by WHO for removal of orthophosphate. However, according to US/EPA, they should not exceed 0.05 mg/L if streams discharge into lakes. Present research showed 0.197 mg/L of phosphate in raw sewage. The concentration of phosphates declined during treatment and showed values of 0.132, 0.083, 0.033, 0.024, and 0.021 mg/L after passing through final polishing step of sand bed filtration (Sb) with subsequent increase in HRT of 4, 8, 12, 16, and 20 days, respectively. In 4-days HRT, only 33.09% of the phosphorus was removed while a significant reduction of 89.35% was observed at HRT of 20 days (Figure 4(a)). Akratos and Tsihrintzis [24] also reported 88.1% phosphorus removal at high HRT of 20 days. This implies that phosphorus removal is not only an issue of porous media but of microbial activity as well. 

The level of sulphates in the domestic wastewater was quite low (0.095 mg/L) as compared to the standard set by WHO. However, it gradually declined during different HRT in HCW (0.057, 0.031, 0.029, and 0.025 mg/L) after 4-, 8-, 12- and 16-days HRT, respectively, until the highest level of reduction was observed in the longest HRT of 20 days, that is, 0.018 mg/L (80.75%) (Figure 4(b)). The removal of sulphate was ascribed to its oxidation by an increase in DO levels during treatment. Krasnits et al. [26] has documented 40% removal of sulphate for a 4-day HRT in a subsurface flow CW. 

The domestic wastewater contained chlorides in an acceptable range of 35.87 mg/L. Chlorides in the domestic wastewater are mainly due to kitchen and laundry waste. In case of chlorides, the removal efficiency of the hybrid system increased by increasing HRT. Treatment with 4-day HRT showed chlorides decline of 57.95% (15.08 mg/L) which reached a maximum level of 84.38% (5.6 mg/L) with 20-day HRT (Figure 4(c)). The decrease of 88.6% in the level of chlorides with HRT of 10 hours was also reported by Valipour et al. [27] in a horizontal subsurface CW.

The concentrations of nitrate and nitrite in the untreated raw sample were 2.83 and 0.059 mg/L, respectively. In the present study, it was observed that initially the concentration of nitrate and nitrite increased 48% and 37% for wastewater respectively, up till 4-day HRT, which suggests the process of nitrification, and then there was drastic decreased after 4 days of treatment which showed denitrification process, that is, conversion of nitrate-nitrogen (NO_3_-N) into the diatomic molecular nitrogen (N_2_) due to which a decrease was observed in EC of domestic wastewater. The most effective reduction was observed at HRT of 20 days; that is, 0.11 mg/L (96.04%) that showed highly significant (*P* < 0.001) treatment (Figure 4(d)). However, Van de Moortel [28] reported similar increase in nitrate-N concentration from 0.5 to 2.7 mg/L at the outflow of their CW. The levels of nitrite decreased gradually with increasing HRT in the hybrid CW. The lowest reduction levels of 0.043 mg/L (27.11%) were observed at HRT of 4 days while the highest removal competence of 0.005 mg/L (91.52%) was exhibited at HRT of 20 days (Figure 4(e)). 

### 3.4. Microbiological Characterization of Wastewater before and after Treatment

#### 3.4.1. Colony-Forming Unit (CFU/mL)

**Figure 5:**
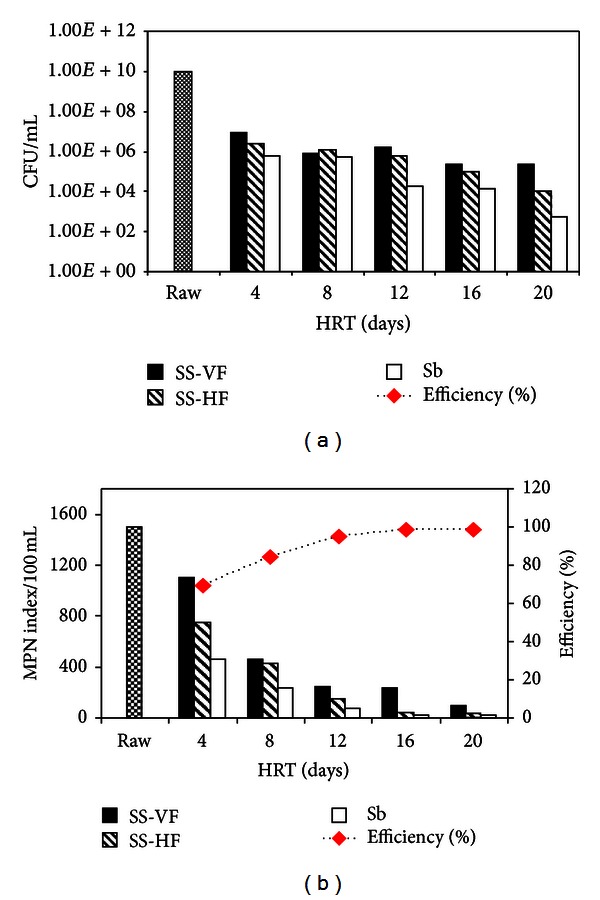
Microbiological analysis of wastewater during treatment under different hydraulic retention times; (a) colony-forming unit (CFU/mL), (b) most probable number (MPN/100 mL).

Figure 5 represents the variation of colony-forming unit (CFU) and MPN index with HRT. The bacterial count for untreated water was found to be 9.3 × 10^9^ CFU/mL. While it shows a decreasing behaviour with increasing HRT (6.37 × 10^5^, 5.29 × 10^5^, 1.79 × 10^5^, 1.4 × 10^4^, and 5.27 × 10^2^ for 4, 8, 12, 16, and 20 days) (Figure 5(a)).

#### 3.4.2. MPN Index of Wastewater

The most probable number (MPN) technique was used for the determination of presence or absence of fecal coliforms in wastewater samples using lactose broth. MPN/100 mL of faecal coliform (*E. coli*), pathogens (*Salmonella, Shigella*), and other coliforms (*Klebsiella, Enterobacter,* and *Citrobacter*) showed that untreated sewage limit was between 150–1500 probably more than 1100. MPN index (as shown in Figure 5(b)) keeps on decreasing by subsequent increase in hydraulic retention times. Samples from three consecutive units (SS-VF, SS-HF, and Sb) of 4 days HRT showed positive results for MPN, that is, 1100, 750 and 460 MPN/100 mL, respectively. With successive increase in hydraulic retention time, MPN index kept on decreasing up to 21/100 mL after passing through final polishing step of sand bed (Sb) filtration with HRT of 20 days. Thus, the hybrid system was very efficient in removing the bacterial contaminants as % reduction was up to 98.6%. Masi and Martinuzzi [29] found efficiencies up to 99.97% with a hybrid (VF + HF) CW treating wastewater from a hotel in Italy. However, Singh et al. [30] found 97.5% removal for a hybrid (HF + VF) CW in Nepal including an anaerobic reactor.

## 4. Conclusion

The HCW system (VF + HF) was proved to be quite effective in reducing BOD_5_, COD, chlorides, sulphates, phosphates as well as faecal coliform (*E. coli*), pathogens (*Salmonella, Shigella*) and other coliforms (*Klebsiella, Enterobacter,* and *Citrobacter*) and increasing DO concentrations in all the treatments. A significant association was found between the percentage removal of contaminants and coliforms with different HRT, and the highest percentage removal was found in HRT of 20 days. A substantial reduction was also observed in SO_4_, PO_4_, NO_3_, and NO_2_ concentrations in the rhizosphere of *Paspalidium flavidum* which indicates the presence of sulfate reducing/oxidizing, phosphate accumulating, nitrifying, and denitrifying bacteria in the biofilm.

## Figures and Tables

**Table 1:** Microscopic and biochemical characterization of isolated bacterial strains from rhizosphere of *Paspalidium flavidum. *

Strains	Microscopy		Fermentation			H_2_S test	NO_3_ test	Indole test	MR test	VP test	Citrate test	Urease test	Catalase test	TSI test	Identified microorganism
	Morphology	Gram staining	Lactose	Dextrose	Sucrose										
1	Rods	−	−	AG	AG	+	+	+	+	−	±	+	+	−	*Proteus* sp.
2	Rods	−	AG	AG	AG	−	+	−	±	±	+	+	+	−	*Klebsiella* sp.
3	Rods	−	AG	AG	A±	−	+	+	+	−	−	−	+	A/NC	*Escherichia coli *
4	Short rods	−	−	A	A±	−	+	±	+	−	−	−	+	K/A, H_2_S	*Shigella* sp.
5	Cocco-bacillus	−	−	−	−	−	−	−	−	−	±	−	+	−	*Alcaligenes* sp.
6	Short rods	−	−	AG±	A±	+	+	−	+	−	+	−	+	K/A, H_2_S	*Salmonella* sp.
7	Rods	−	AG	AG	AG	−	+	−	−	+	+	−	+	K/A	*Enterobacter* sp.
8	Rods	+	−	A	A	−	+	−	−	±	−	−	+	A/NC	*Bacillus* sp.
9	Rods	−	−	−	−	−	+	−	−	−	+	−	+	−	*Pseudomonas* sp.
10	Cocci	+	−	−	−	−	±	−	−	−	−	+	+	K/NC	*Micrococcus* sp.
11	Cocci	+	A	A	A	−	+	−	+	±	−	−	+	A/A	*Staphylococcus* sp.

Key: AG: acid and gas; +: positive; −: negative; ±: variable reaction; A: acid production; K: alkaline reaction; NC: no change; H_2_S: Sulfur reduction; K/A: Red/yellow; K/NC: Red/no color change; K/A, H_2_S: red/yellow with bubble and black precipitate; A/NC: acid/no color change; A/A: yellow/yellow.
